# Supplementary material for: Origin, Strength, and Speed of the Nonlinear Optical Response of Transparent Conducting Oxides to Single‐Cycle Optical Pulses
Source: Nanophotonics. 2026 Aug 28;15(17):e70272. doi: 10.1002/nap2.70272 (PMC13672169; doi:10.1002/nap2.70272)
Supplement: Supplementary file 1 [file NAP2-15-e70272-s001.pdf]

Supporting Information for Origin, strength, and speed of the nonlinear optical response of transparent conducting oxides to single-cycle optical pulses

*Ieng-Wai Un** *Subhajit Sarkar* *Yonatan Sivan*

Ieng-Wai Un
Key Laboratory of Atomic and Subatomic Structure and Quantum Control (Ministry of Education), Guangdong Basic Research Center of Excellence for Structure and Fundamental Interactions of Matter, School of Physics, South China Normal University, Guangzhou 510006, China.
Guangdong Provincial Key Laboratory of Quantum Engineering and Quantum Materials, Guangdong-Hong Kong Joint Laboratory of Quantum Matter, South China Normal University, Guangzhou 510006, China.
Email Address: iengwai@m.scnu.edu.cn
Subhajit Sarkar
Department of Physics, School of Natural Sciences, Shiv Nadar Institution of Eminence Deemed to be University, Delhi-NCR, NH91, Tehsil Dadri, Greater Noida, Uttar Pradesh 201314, India.
Yonatan Sivan
School of Electrical and Computer Engineering, Ben-Gurion University of the Negev, Beer Sheva, 8410501, Israel.

S1 Density matrix formulation for single-cycle pulse interaction with ITO

The density matrix formulation for the interactions of an intense single-cycle pulse with a TCO NP was shown in Ref. [1] to be given by

$$\begin{cases} \dfrac{\partial f_{\mathbf{k}}(t)}{\partial t} = -\dfrac{i}{\hbar}E_{\mathrm{NP}}(t)\displaystyle\sum_{\mathbf{k}'}\left[d_{\mathbf{k}'\mathbf{k}}\rho_{\mathbf{k}\mathbf{k}'}(t) - d_{\mathbf{k}\mathbf{k}'}\rho_{\mathbf{k}'\mathbf{k}}(t)\right] + \left(\dfrac{\partial f_{\mathbf{k}}}{\partial t}\right)_{\text{e-e}} + \left(\dfrac{\partial f_{\mathbf{k}}}{\partial t}\right)_{\text{e-ph}} + \left(\dfrac{\partial f_{\mathbf{k}}}{\partial t}\right)_{\text{e-imp}} & \text{(S1a)} \\ \dfrac{\partial \rho_{\mathbf{k}\mathbf{k}'}(t)}{\partial t} = -\eta_{\mathbf{k}\mathbf{k}'}\left(\rho_{\mathbf{k}\mathbf{k}'} - h_{\mathbf{k}\mathbf{k}'}\rho_{\mathbf{k}'\mathbf{k}}\right) - \dfrac{i}{\hbar}\left(\mathcal{E}_{\mathbf{k}} - \mathcal{E}_{\mathbf{k}'}\right)\rho_{\mathbf{k}\mathbf{k}'} + \dfrac{i}{\hbar}E_{\mathrm{NP}}(t)d_{\mathbf{k}\mathbf{k}'}\left[f_{\mathbf{k}'}(t) - f_{\mathbf{k}}(t)\right], & \text{(S1b)} \end{cases}$$

where $f_{\mathbf{k}}(t)$ and $\rho_{\mathbf{k}\mathbf{k}'}(t)$ are, respectively, the diagonal and off-diagonal elements of the density matrix; they represent the populations of each momentum state $\mathbf{k}$ and the coherence between every pair of them; E is the local electric field, $d_{\mathbf{k}\mathbf{k}'}$ are the transition dipole matrix elements [2, 3], and $\mathcal{E}_k$ is the electron energy. On the right-hand side of Equation (S1a), the first term represents the electron-photon (e-photon) interaction; the last three terms, $(\partial f_{\mathbf{k}}/\partial t)_{\text{e-e}}$, $(\partial f_{\mathbf{k}}/\partial t)_{\text{e-ph}}$, and $(\partial f_{\mathbf{k}}/\partial t)_{\text{e-imp}}$ represent the rates of change of the electronic population at momentum state $\mathbf{k}$ arising from electron–electron (e-e), electron–phonon (e-ph), and electron–impurity (e-imp) scattering processes, respectively. The e-e collision term is derived from the screened Coulomb interaction following Fermi's golden rule [4]

$$\left(\frac{\partial f_{\mathbf{k}}}{\partial t}\right)_{\text{e-e}} = -\frac{3}{4}\frac{2\pi}{\hbar}\frac{1}{2}\sum_{\mathbf{k}_1,\mathbf{k}_2,\mathbf{k}_3}|M(\mathbf{q})|^2\delta_{\mathbf{k}+\mathbf{k}_1,\mathbf{k}_2+\mathbf{k}_3}\delta(\mathcal{E}+\mathcal{E}_1-\mathcal{E}_2-\mathcal{E}_3)$$
$$\left[(1-f_{\mathbf{k}_3})(1-f_{\mathbf{k}_2})f_{\mathbf{k}_1}f_{\mathbf{k}} - (1-f_{\mathbf{k}})(1-f_{\mathbf{k}_1})f_{\mathbf{k}_2}f_{\mathbf{k}_3}\right], \quad \text{(S2)}$$

where $M(\mathbf{q})$ is the Fourier transform of the Thomas-Fermi screened Coulomb potential[5]

$$M(\mathbf{q}) = \frac{e^2}{\varepsilon_0\varepsilon_\infty(q^2+q_{\mathrm{TF}}^2)}, \quad \text{(S3)}$$

and q_{TF} is the inverse screening length given by [6][1]

$$q_{\mathrm{TF}}^2 = -\frac{e^2}{\varepsilon_0 \varepsilon_\infty} \sum_{\mathbf{k}} \frac{\partial f_{\mathbf{k}}}{\partial \mathcal{E}_{\mathbf{k}}} \tag{S4}$$

The *e-ph* collision term via the deformation potential is given by [4]

$$\begin{aligned}\left(\frac{\partial f_{\mathbf{k}}}{\partial t}\right)_{\text{e-ph}} = \sum_{\mathbf{q}} \frac{\pi D^2 q^2}{\rho V \omega_{\mathrm{ph}}^{\mathbf{q}}} \Big\{ & \left[(n_{\mathrm{ph}}^{\mathbf{q}}+1)(1-f_{\mathbf{k}}) f_{\mathbf{k}+\mathbf{q}} - n_{\mathrm{ph}}^{\mathbf{q}}(1-f_{\mathbf{k}+\mathbf{q}}) f_{\mathbf{k}}\right] \delta(\mathcal{E}_{\mathbf{k}+\mathbf{q}} - \mathcal{E}_{\mathbf{k}} - \hbar\omega_{\mathrm{ph}}^{\mathbf{q}}) \\ & - \left[(n_{\mathrm{ph}}^{\mathbf{q}}+1)(1-f_{\mathbf{k}-\mathbf{q}}) f_{\mathbf{k}} - n_{\mathrm{ph}}^{\mathbf{q}}(1-f_{\mathbf{k}}) f_{\mathbf{k}-\mathbf{q}}\right] \delta(\mathcal{E}_{\mathbf{k}-\mathbf{q}} - \mathcal{E}_{\mathbf{k}} + \hbar\omega_{\mathrm{ph}}^{\mathbf{q}}) \Big\},\end{aligned} \tag{S5}$$

where $\mathbf{q}$ is the phonon momentum, and the phonon dispersion is approximated as linear, $\hbar\omega_{\mathrm{ph}}^{\mathbf{q}} = \hbar v_{\mathrm{ph}} q$, with v_{ph} being the sound velocity. The parameters ρ and D denote the material density and the deformation potential, respectively. The phonon occupation number $n_{\mathrm{ph}}^{\mathbf{q}}(\hbar\omega_{\mathrm{ph}}^{\mathbf{q}}, T_{\mathrm{ph}}) = \left(e^{\hbar\omega_{\mathrm{ph}}^{\mathbf{q}}/k_B T_{\mathrm{ph}}} - 1\right)^{-1}$ follows the Bose-Einstein statistics characterized by the phonon (lattice) temperature T_{ph}. In addition, the phonon density of states is assumed to vanish for phonon energies exceeding the Debye energy $\mathcal{E}_D$. Finally, the e-imp collision term is modeled using the Brooks-Herring approach via a screened Coulomb interaction,

$$\left(\frac{\partial f_{\mathbf{k}}}{\partial t}\right)_{\text{e-imp}} = \frac{2\pi}{\hbar} \frac{n_{\mathrm{imp}}}{V} \sum_{\mathbf{k}'} \left[\frac{Ze^2}{\varepsilon_0 \varepsilon_b} \frac{1}{(|\mathbf{k}-\mathbf{k}'|^2 + q_{\mathrm{TF}}^2)}\right]^2 \left[f_{\mathbf{k}'}(1-f_{\mathbf{k}}) - f_{\mathbf{k}}(1-f_{\mathbf{k}'})\right] \delta(\mathcal{E}_{\mathbf{k}} - \mathcal{E}_{\mathbf{k}'}). \tag{S6}$$

Here, n_{imp} is the impurity concentration and Z is the impurity charge. In Equation(S1b), $h_{\mathbf{k}\mathbf{k}'}$ is a unitless phase factor such that $h_{\mathbf{k}\mathbf{k}'} d_{\mathbf{k}'\mathbf{k}} = d_{\mathbf{k}\mathbf{k}'}$. This relation guarantees charge-density continuity (see details in Refs. [8, 9]). $\eta_{\mathbf{k}\mathbf{k}'}(t) = (\eta_{\mathbf{k}}(t) + \eta_{\mathbf{k}'}(t))/2$ represents the total decoherence rate of electrons at different states, including *e-e*, *e-ph*, and *e-imp* collisions, namely, $\eta_{\mathbf{k}} = \eta_{\text{e-e},\mathbf{k}} + \eta_{\text{e-ph},\mathbf{k}} + \eta_{\text{e-imp},\mathbf{k}}$. Each contribution is obtained from the functional derivative of the corresponding relaxation term (Equations (S2), (S5) and (S6)) with respect to the distribution function $f_{\mathbf{k}}$ [10],

$$\eta_{\text{e-e},\mathbf{k}} = \frac{3}{4} \frac{2\pi}{\hbar} \sum_{\mathbf{k}_1,\mathbf{k}_2,\mathbf{k}_3} |M(\mathbf{q})|^2 \delta_{\mathbf{k}+\mathbf{k}_1,\mathbf{k}_2+\mathbf{k}_3} \delta(\mathcal{E} + \mathcal{E}_1 - \mathcal{E}_2 - \mathcal{E}_3) \left[(1-f_{\mathbf{k}_3})(1-f_{\mathbf{k}_2}) f_{\mathbf{k}_1} + f_{\mathbf{k}_3} f_{\mathbf{k}_2}(1-f_{\mathbf{k}_1})\right], \tag{S7}$$

$$\eta_{\text{e-ph},\mathbf{k}} = \sum_{\mathbf{q}} \frac{\pi D^2 q^2}{\rho V \omega_{\mathrm{ph}}^{\mathbf{q}}} \Big[\left(n_{\mathrm{ph}}^{\mathbf{q}} + f_{\mathbf{k}+\mathbf{q}}\right) \delta(\mathcal{E}_{\mathbf{k}+\mathbf{q}} - \mathcal{E}_{\mathbf{k}} - \hbar\omega_{\mathrm{ph}}^{\mathbf{q}}) + \left(n_{\mathrm{ph}}^{\mathbf{q}} + 1 - f_{\mathbf{k}-\mathbf{q}}\right) \delta(\mathcal{E}_{\mathbf{k}-\mathbf{q}} - \mathcal{E}_{\mathbf{k}} + \hbar\omega_{\mathrm{ph}}^{\mathbf{q}}) \Big], \tag{S8}$$

and

$$\eta_{\text{e-imp},\mathbf{k}} = \frac{2\pi}{\hbar} \frac{n_{\mathrm{imp}}}{(2\pi)^3} \left(\frac{Ze^2}{\varepsilon_0 \varepsilon_b}\right)^2 \frac{\pi}{k^2} \frac{dk}{d\mathcal{E}} \left[\left(1 - \frac{q_{\mathrm{TF}}^2}{2k^2}\right)^2 - 1\right]^{-1}. \tag{S9}$$

The solution of Equation (S1) enables the evaluation of the total polarization density, which is then coupled self-consistently to Maxwell's equations. The total polarization is decomposed into non-dispersive and dispersive components. The non-dispersive component accounts for the response of bound electrons and, given our focus on near-infrared frequencies, is assumed to remain unaffected by optical excitation. It is characterized by a constant susceptibility, $\varepsilon_\infty - 1$, where ε_∞ denotes the infinite-frequency permittivity. The dispersive component, denoted by $\mathbf{P}_{\mathrm{NP}}$, is obtained by taking the trace of the matrix multiplication of the density matrix (solution of Equation (S1)) and the dipole transition matrix. Explicitly,

$$P_{\mathrm{NP}}(t) = \frac{2}{V} \sum_{\mathbf{k},\mathbf{k}'} d_{\mathbf{k}'\mathbf{k}} \rho_{\mathbf{k}\mathbf{k}'}(t). \tag{S10}$$

[1] Note that the expression that appeared in our earlier work (Equation (15) in [7]) had erroneously included also a factor of $4\pi^3$.

In the weak-field and continuous wave limit, the Fourier transform of $P(t)$ (Equation (S10)) recovers the Drude susceptibility multiplied by the Fourier-transformed local electric field. The corresponding permittivity (Figure 1(b)) can therefore be expressed as (see also Ref. [11])

$$\varepsilon_{\text{lin}}(\omega) = \varepsilon_\infty + \sum_{\mathbf{k},\mathbf{k}'} \frac{2|d_{\mathbf{k},\mathbf{k}'}|^2(\mathcal{E}_\mathbf{k} - \mathcal{E}_{\mathbf{k}'})(f^{\text{FD}}_{\mathbf{k}'} - f^{\text{FD}}_\mathbf{k})}{(\mathcal{E}_\mathbf{k} - \mathcal{E}_{\mathbf{k}'})^2 - (\hbar\omega)^2 - 2i\hbar^2\omega\eta^{\text{FD}}_{\mathbf{k},\mathbf{k}'}}. \tag{S11}$$

Here, $f^{\text{FD}}_\mathbf{k} \equiv f^{\text{FD}}(\mathcal{E}_\mathbf{k}, \mu(300\text{ K}), 300\text{ K})$ and $\eta^{\text{FD}}_{\mathbf{k},\mathbf{k}'}$ denote the thermalized Fermi-Dirac electron distribution and the corresponding collision rate, respectively. By replacing the electron distribution $f^{\text{FD}}_\mathbf{k}$ and $\eta_{\mathbf{k},\mathbf{k}'}$ with the time-dependent electron distribution obtained from Equation (S1a) and the corresponding collision rates, Equation (S11) yields the "instantaneous permittivity" within the adiabatic non-thermal permittivity model (ANTH$\mathcal{E}$M) [7]

$$\varepsilon_{\text{ANTH}\mathcal{E}\text{M}}(t;\omega) = \varepsilon_\infty + \sum_{\mathbf{k},\mathbf{k}'} \frac{2|d_{\mathbf{k},\mathbf{k}'}|^2(\mathcal{E}_\mathbf{k} - \mathcal{E}_{\mathbf{k}'})(f_{\mathbf{k}'}(t) - f_\mathbf{k}(t))}{(\mathcal{E}_\mathbf{k} - \mathcal{E}_{\mathbf{k}'})^2 - (\hbar\omega)^2 - 2i\hbar^2\omega\eta_{\mathbf{k},\mathbf{k}'}(t)}. \tag{S12}$$

The e-photon (the first term on the right-hand side of Equation (S1a)) and the polarization (Equation (S10)) can be divided into the stimulated emission and absorption components by splitting the coherence as $\rho_{\mathbf{k}\mathbf{k}'} = \rho_{\text{abs},\mathbf{k}\mathbf{k}'} + \rho_{\text{em},\mathbf{k}\mathbf{k}'}$. This yields

$$\left(\frac{\partial f_\mathbf{k}}{\partial t}\right)_{e-\text{photon}} = -\frac{i}{\hbar}E_{\text{NP}}(t)\sum_{\mathbf{k}'}\Big[(d_{\mathbf{k}'\mathbf{k}}\rho_{\text{abs},\mathbf{k}\mathbf{k}'} - d_{\mathbf{k}\mathbf{k}'}\rho_{\text{abs},\mathbf{k}'\mathbf{k}}) + (d_{\mathbf{k}'\mathbf{k}}\rho_{\text{em},\mathbf{k}\mathbf{k}'} - d_{\mathbf{k}\mathbf{k}'}\rho_{\text{em},\mathbf{k}'\mathbf{k}})\Big] \tag{S13}$$

and

$$P_{\text{NP}}(t) = P_{\text{NP,abs}}(t) + P_{\text{NP,em}}(t) = \frac{2}{V}\sum_{\mathbf{k},\mathbf{k}'}\left[d_{\mathbf{k}'\mathbf{k}}\rho_{\text{abs},\mathbf{k}\mathbf{k}'} + d_{\mathbf{k}'\mathbf{k}}\rho_{\text{em},\mathbf{k}\mathbf{k}'}\right]. \tag{S14}$$

Here, $\rho_{\text{em},\mathbf{k}\mathbf{k}'}$ and $\rho_{\text{abs},\mathbf{k}\mathbf{k}'}$ denote the coherences associated with stimulated emission and absorption, respectively. To derive their governing equations, we decompose the occupation difference in Equation (S11) as

$$\begin{aligned} f_{\mathbf{k}'} - f_\mathbf{k} = f_{\mathbf{k}'}(1-f_\mathbf{k})\Theta(\mathcal{E}_\mathbf{k} - \mathcal{E}_{\mathbf{k}'}) - f_\mathbf{k}(1-f_{\mathbf{k}'})\Theta(\mathcal{E}_{\mathbf{k}'} - \mathcal{E}_\mathbf{k}) \\ - f_\mathbf{k}(1-f_{\mathbf{k}'})\Theta(\mathcal{E}_{\mathbf{k}'} - \mathcal{E}_\mathbf{k}) + f_{\mathbf{k}'}(1-f_\mathbf{k})\Theta(\mathcal{E}_{\mathbf{k}'} - \mathcal{E}_\mathbf{k}), \end{aligned} \tag{S15}$$

where Θ is the Heaviside step function. The first (last) two terms represent upward (absorption) and downward (emission) transitions. This leads to the following equations of motion:

$$\begin{cases} \dfrac{\partial \rho_{\text{abs},\mathbf{k}\mathbf{k}'}(t)}{\partial t} = -\eta_{\mathbf{k}\mathbf{k}'}\left(\rho_{\text{abs},\mathbf{k}\mathbf{k}'} - h_{\mathbf{k}\mathbf{k}'}\rho_{\text{abs},\mathbf{k}'\mathbf{k}}\right) - \dfrac{i}{\hbar}\left(\mathcal{E}_\mathbf{k} - \mathcal{E}_{\mathbf{k}'}\right)\rho_{\text{abs}\mathbf{k}\mathbf{k}'} \\ \qquad + \dfrac{i}{\hbar}E_{\text{NP}}(t)d_{\mathbf{k}\mathbf{k}'}\left[f_{\mathbf{k}'}(1-f_\mathbf{k})\Theta(\mathcal{E}_\mathbf{k} - \mathcal{E}_{\mathbf{k}'}) - f_\mathbf{k}(1-f_{\mathbf{k}'})\Theta(\mathcal{E}_{\mathbf{k}'} - \mathcal{E}_\mathbf{k})\right], & \text{(S16a)} \\ \dfrac{\partial \rho_{\text{em},\mathbf{k}\mathbf{k}'}(t)}{\partial t} = -\eta_{\mathbf{k}\mathbf{k}'}\left(\rho_{\text{em},\mathbf{k}\mathbf{k}'} - h_{\mathbf{k}\mathbf{k}'}\rho_{\text{em},\mathbf{k}'\mathbf{k}}\right) - \dfrac{i}{\hbar}\left(\mathcal{E}_\mathbf{k} - \mathcal{E}_{\mathbf{k}'}\right)\rho_{\text{em},\mathbf{k}\mathbf{k}'} \\ \qquad + \dfrac{i}{\hbar}E_{\text{NP}}(t)d_{\mathbf{k}\mathbf{k}'}\left[-f_\mathbf{k}(1-f_{\mathbf{k}'})\Theta(\mathcal{E}_{\mathbf{k}'} - \mathcal{E}_\mathbf{k}) + f_{\mathbf{k}'}(1-f_\mathbf{k})\Theta(\mathcal{E}_{\mathbf{k}'} - \mathcal{E}_\mathbf{k})\right], & \text{(S16b)} \end{cases}$$

A similar decomposition can be applied to the linear permittivity (Equation (S11)), as well as to the "instantaneous permittivity" defined in Equation (S12).

To reduce the dimensionality of the problem and facilitate numerical implementation, we reformulate the kinetic equations from momentum space to energy space, see details in Ref. [1]. Specifically, the summation over momentum states in Equation (S1b) is replaced by an integral according to

$$\frac{V}{(2\pi)^3}\int d^3k' = \frac{V}{(2\pi)^3}\int \pi^2\rho_e(\mathcal{E}_{\mathbf{k}'})d\mathcal{E}_{\mathbf{k}'}d\Omega_{\mathbf{k}'},$$

where $\Omega_{\mathbf{k}'}$ denotes the solid-angle in momentum space. The angular integration over $\Omega_{\mathbf{k}'}$ is then carried out explicitly, yielding an energy-resolved formulation. The same transformation is applied to the collision terms in Equations (S2), (S5) and (S6), and to the polarization density Equation (S10).

Table S1: Parameters used in the simulations.

Physical quantity	Symbol	Value
non-parabolicity (bottom of the conduction band)	C	0.4191 eV^{-1} [12]
effective mass	m_e^*	0.3964 m_e [12]
Fermi energy	$\mathcal{E}_F$	0.8793 eV
electron desnity	n_e	$\sim 1.5 \times 10^{27}$ m^{-3}
deformation potential	D	8.6 eV [a]
material density	ρ	7120 $\mathrm{kg/m}^2$ [14]
sound velocity	v_{ph}	6400 m/s [14]
Debye energy	$\mathcal{E}_D$	0.06 eV [14]
impurity concentration	n_{imp}	0.05 nm^{-3} [13]
impurity charge	Z_{imp}	1 [13]

[a] This value of the deformation potential is obtained by fitting the ITO permittivity at the bulk limit and at room temperature with that measured experimentally [13].

S2 Derivation of the generalized Fermi Liquid theory expression for *e*-*e* interactions at high temperature and far-from-equilibrium conditions

The electron-electron (e-e) scattering rate can be obtained by taking the functional derivative of the corresponding distribution change rate [10, 15]

$$\eta_{\text{e-e}}(\mathcal{E}) = \frac{3}{4}\frac{2\pi}{\hbar}\frac{(2\pi)^2}{2}\frac{V}{(2\pi)^6}\left(\frac{m_e^*}{\hbar^2}\right)^3 \int_{\mathcal{E}_{\min}}^{\mathcal{E}_{\max}} d\mathcal{E}_1 d\mathcal{E}_2 d\mathcal{E}_3 \frac{1}{k}\left[\int_{q_{\min}}^{q_{\max}} dq |M(q)|^2\right] \delta(\mathcal{E}+\mathcal{E}_1-\mathcal{E}_2-\mathcal{E}_3)$$
$$[(1-f(\mathcal{E}_3))(1-f(\mathcal{E}_2))f(\mathcal{E}_1)+(1-f(\mathcal{E}_1))f(\mathcal{E}_2)f(\mathcal{E}_3)]\,(1+2C\mathcal{E}_1)(1+2C\mathcal{E}_2)(1+2C\mathcal{E}_3), \quad \text{(S17)}$$

where $\mathcal{E}$ is the electron energy, m_e^* is the Kane mass (equivalently, the effective mass at the band minimum), C is the non-parabolicity, $\hbar k$ is the electron momentum satisfying the $\mathcal{E}$-k relation $(\hbar k)^2 = 2m_e^*\mathcal{E}(1+C\mathcal{E})$, $\mathcal{E}_{\min}$ and $\mathcal{E}_{\max}$ are the bottom and top conduction band-edge energies, $q_{\max} = \min(k_1+k_3, k+k_2)$, $q_{\min} = \max(|k_1-k_3|, |k-k_2|)$ and $|M(q)|^2$ is the $e-e$ scattering matrix element. Here, the factor $(1+2C\mathcal{E}_1)(1+2C\mathcal{E}_2)(1+2C\mathcal{E}_3)$ arises from the substitution $k = k(\mathcal{E})$ when transforming the scattering rate (S7) from momentum to energy space. Specifically, in Equation (S7) the Kronecker delta enforcing momentum conservation $\delta_{\mathbf{k}+\mathbf{k}_1,\mathbf{k}_2+\mathbf{k}_3}$ is replaced in the continuum limit by $\frac{(2\pi)^3}{V}\delta(\mathbf{k}+\mathbf{k}_1-\mathbf{k}_2-\mathbf{k}_3)$. At the same time, the summation over momenta is converted into integrals according to $\sum_{\mathbf{k}_1,\mathbf{k}_2,\mathbf{k}_3} \to \frac{V^3}{(2\pi)^9}\iiint d\mathbf{k}_1 d\mathbf{k}_2 d\mathbf{k}_3$ which, in spherical coordinates, becomes $\frac{V^3}{(2\pi)^9}\int k_1^2 dk_1 d\Omega_{\mathbf{k}_1}\int k_2^2 dk_2 d\Omega_{\mathbf{k}_2}\int k_3^2 dk_3 d\Omega_{\mathbf{k}_3}$. Integrating the delta function $\delta(\mathbf{k}+\mathbf{k}_1-\mathbf{k}_2-\mathbf{k}_3)$ over the solid angles of $\mathbf{k}_1$, $\mathbf{k}_2$ and $\mathbf{k}_3$ yields a factor proportional to $\int dq\frac{1}{k_1k_2k_3}$, where $q = |\mathbf{k}_2-\mathbf{k}| = |\mathbf{k}_1-\mathbf{k}_3|$. Combining this result with $k_1^2dk_1k_2^2dk_2k_3^2dk_3$ gives $k_1dk_1k_2dk_2k_3dk_3$. Upon transforming variables from momentum to energy, this becomes $k_1dk_1k_2dk_2k_3dk_3 \propto \frac{d(k_1^2)}{d\mathcal{E}_1}\frac{d(k_2^2)}{d\mathcal{E}_2}\frac{d(k_3^2)}{d\mathcal{E}_3}d\mathcal{E}_1d\mathcal{E}_2d\mathcal{E}_3$. For the Kane non-parabolic dispersion considered here, $k_n^2 \propto \mathcal{E}_n(1+C\mathcal{E}_n)$, $n = 1,2,3$, so that $\frac{d(k_n^2)}{d\mathcal{E}_n} \propto (1+2C\mathcal{E}_n)$. Consequently, the Jacobian of the transformation produces the factor $(1+2C\mathcal{E}_1)(1+2C\mathcal{E}_2)(1+2C\mathcal{E}_3)$.

The q-integration of the scattering matrix element $|M|^2$ (Equation (S3)) gives

$$\int_{q_{\rm min}}^{q_{\rm max}} dq|M(q)|^2 = \left(\frac{e^2}{\varepsilon_0\varepsilon_b^0}\right)^2 \frac{1}{2q_{\rm TF}^3}\left[\frac{qq_{\rm TF}}{q^2+q_{\rm TF}^2}+\tan^{-1}\left(\frac{q}{q_{\rm TF}}\right)\right]_{q_{\rm min}}^{q_{\rm max}}. \tag{S18}$$

Combining Equations (S17) and (S18) gives

$$\begin{aligned}\eta_{\text{e-e}}(\mathcal{E}) = \frac{3}{4}\frac{2\pi}{\hbar}\frac{(2\pi)^2}{2}\frac{1}{(2\pi)^6}\left(\frac{m_e^*}{\hbar^2}\right)^3\left(\frac{e^2}{\varepsilon_0\varepsilon_b^0}\right)^2\frac{1}{2q_{\rm TF}^3}\int d\mathcal{E}_1 d\mathcal{E}_2 d\mathcal{E}_3\frac{1}{k}\\ \left[\frac{qq_{\rm TF}}{q^2+q_{\rm TF}^2}+\tan^{-1}\left(\frac{q}{q_{\rm TF}}\right)\right]_{q_{\rm min}}^{q_{\rm max}}\delta(\mathcal{E}+\mathcal{E}_1-\mathcal{E}_2-\mathcal{E}_3)(1+2C\mathcal{E}_1)(1+2C\mathcal{E}_2)\\ (1+2C\mathcal{E}_3)\left[(1-f(\mathcal{E}_3))(1-f(\mathcal{E}_2))f(\mathcal{E}_1)+(1-f(\mathcal{E}_1))f(\mathcal{E}_2)f(\mathcal{E}_3)\right].\end{aligned} \tag{S19}$$

The Fermi Liquid Theory (FLT) proceeds by neglecting contributions from the non-thermal distribution by approximating the electron distribution function $f(\mathcal{E})$ in Equation (S19) by a thermalized Fermi-Dirac distribution, $f(\mathcal{E}) \approx f^{\rm FD}(\mathcal{E},\mu(\tilde{T}_e),\tilde{T}_e)$, where $\mu(\tilde{T}_e)$ is the chemical potential at the effective electron temperature $\tilde{T}_e$. This temperature is chosen such that the total energy of the approximated thermal distribution matches that of the original distribution, i.e.,

$$\int_{\mathcal{E}_{\rm min}}^{\mathcal{E}_{\rm max}} d\mathcal{E} f(\mathcal{E})\mathcal{E}\rho_e(\mathcal{E}) = \int_{\mathcal{E}_{\rm min}}^{\mathcal{E}_{\rm max}} d\mathcal{E} f^{\rm FD}(\mathcal{E},\mu(\tilde{T}_e),\tilde{T}_e)\mathcal{E}\rho_e(\mathcal{E}). \tag{S20}$$

With this approximation, the $e-e$ scattering rate becomes

$$\begin{aligned}\eta^T_{\text{e-e}}(\mathcal{E}) = \frac{3}{4}\frac{2\pi}{\hbar}\frac{(2\pi)^2}{2}\frac{1}{(2\pi)^6}\left(\frac{m_e^*}{\hbar^2}\right)^3\left(\frac{e^2}{\varepsilon_0\varepsilon_b^0}\right)^2\frac{1}{2q_{\rm TF}^3}\int_{\mathcal{E}_{\rm min}}^{\mathcal{E}_{\rm max}} d\mathcal{E}_1 d\mathcal{E}_2 d\mathcal{E}_3\\ \frac{1}{k}\left[\frac{qq_{\rm TF}}{q^2+q_{\rm TF}^2}+\tan^{-1}\left(\frac{q}{q_{\rm TF}}\right)\right]_{q_{\rm min}}^{q_{\rm max}}\delta(\mathcal{E}+\mathcal{E}_1-\mathcal{E}_2-\mathcal{E}_3)(1+2C\mathcal{E}_1)(1+2C\mathcal{E}_2)(1+2C\mathcal{E}_3)\\ \Big[(1-f^{\rm FD}(\mathcal{E}_3,\mu(\tilde{T}_e),\tilde{T}_e))(1-f^{\rm FD}(\mathcal{E}_2,\mu(\tilde{T}_e),\tilde{T}_e))f^{\rm FD}(\mathcal{E}_1,\mu(\tilde{T}_e),\tilde{T}_e)\\ +(1-f^{\rm FD}(\mathcal{E}_1,\mu(\tilde{T}_e),\tilde{T}_e))f^{\rm FD}(\mathcal{E}_2,\mu(\tilde{T}_e),\tilde{T}_e))f^{\rm FD}(\mathcal{E}_3,\mu(\tilde{T}_e),\tilde{T}_e))\Big],\end{aligned} \tag{S21}$$

and the explicit expression for the product of the Fermi-Dirac functions in the integrand is

$$\begin{aligned}\Big[(1-f^{\rm FD}(\mathcal{E}_3,\mu(\tilde{T}_e),\tilde{T}_e))(1-f^{\rm FD}(\mathcal{E}_2,\mu(\tilde{T}_e),\tilde{T}_e))f^{\rm FD}(\mathcal{E}_1,\mu(\tilde{T}_e),\tilde{T}_e)\\ +(1-f^{\rm FD}(\mathcal{E}_1,\mu(\tilde{T}_e),\tilde{T}_e))f^{\rm FD}(\mathcal{E}_2,\mu(\tilde{T}_e),\tilde{T}_e))f^{\rm FD}(\mathcal{E}_3,\mu(\tilde{T}_e),\tilde{T}_e))\Big]\\ = \cosh\left(\frac{\mathcal{E}-\mu(\tilde{T}_e)}{2k_B\tilde{T}_e}\right)\left[\cosh\left(\frac{\mathcal{E}_1-\mu(\tilde{T}_e)}{2k_B\tilde{T}_e}\right)\cosh\left(\frac{\mathcal{E}_2-\mu(\tilde{T}_e)}{2k_B\tilde{T}_e}\right)\cosh\left(\frac{\mathcal{E}+\mathcal{E}_1-\mathcal{E}_2-\mu(\tilde{T}_e)}{2k_B\tilde{T}_e}\right)\right]^{-1}.\end{aligned} \tag{S22}$$

Now, unlike the conventional Fermi-liquid approximation, where the entire integrand is expanded in powers of $(\mathcal{E}-\mu\tilde{T}_e)/(k_B\tilde{T}_e)$, we expand only the product of Fermi-Dirac functions in the integrand (Equation (S22)) up to the second order, while keeping the $q-$integrated scattering matrix element (S18) exact. Then, we

obtain the approximation

$$\begin{aligned}
&\Big[(1-f^{\mathrm{FD}}(\mathcal{E}_3,\mu(\tilde{T}_e),\tilde{T}_e))(1-f^{\mathrm{FD}}(\mathcal{E}_2,\mu(\tilde{T}_e),\tilde{T}_e))f^{\mathrm{FD}}(\mathcal{E}_1,\mu(\tilde{T}_e),\tilde{T}_e)\\
&\qquad + (1-f^{\mathrm{FD}}(\mathcal{E}_1,\mu(\tilde{T}_e),\tilde{T}_e))f^{\mathrm{FD}}(\mathcal{E}_2,\mu(\tilde{T}_e),\tilde{T}_e))f^{\mathrm{FD}}(\mathcal{E}_3,\mu(\tilde{T}_e),\tilde{T}_e))\Big]\\
&\approx \left[1-\frac{1}{2}\tanh\left(\frac{\mathcal{E}_1-\mathcal{E}_2}{k_B\tilde{T}_e}\right)\frac{\mathcal{E}-\mu(\tilde{T}_e)}{k_B\tilde{T}_e}+\frac{1}{4}\tanh^2\left(\frac{\mathcal{E}_1-\mathcal{E}_2}{k_B\tilde{T}_e}\right)\left(\frac{\mathcal{E}-\mu(\tilde{T}_e)}{k_B\tilde{T}_e}\right)^2\right]\\
&\qquad \left[\cosh\left(\frac{\mathcal{E}_1-\mu(\tilde{T}_e)}{2k_B\tilde{T}_e}\right)\cosh\left(\frac{\mathcal{E}_2-\mu(\tilde{T}_e)}{2k_B\tilde{T}_e}\right)\cosh\left(\frac{\mathcal{E}_1-\mathcal{E}_2}{2k_B\tilde{T}_e}\right)\right]^{-1}. \qquad \text{(S23)}
\end{aligned}$$

Substituting Equation (S23) into Equation (S21) and performing the integrals, we find a compact approximate form for the relaxation rate:

$$\eta^T_{\text{e-e}}(\mathcal{E},\tilde{T}_e)\approx K(\tilde{T}_e)\left[A_0(\mathcal{E},\tilde{T}_e)(k_B\tilde{T}_e)^2+A_1(\mathcal{E},\tilde{T}_e)(k_B\tilde{T}_e)\left(\mathcal{E}-\mu(\tilde{T}_e)\right)+A_2(\mathcal{E},\tilde{T}_e)\left(\mathcal{E}-\mu(\tilde{T}_e)\right)^2\right], \qquad \text{(S24)}$$

where the prefactor $K(\tilde{T}_e)$ is the same as Equation (19) in [10], i.e.,

$$K(\tilde{T}_e)=\frac{3}{32\hbar}\left(\frac{m_e^*}{\hbar^2 q_{\mathrm{TF}}(\tilde{T}_e)}\right)^3\left(\frac{e^2}{\varepsilon_0\varepsilon_b}\right)^2\frac{(1+2C\mu(\tilde{T}_e))^3}{k(\mu(\tilde{T}_e))}\left[\frac{2k_\mu(\tilde{T}_e)q_{\mathrm{TF}}(\tilde{T}_e)}{4k_\mu^2(\tilde{T}_e)+q_{\mathrm{TF}}^2(\tilde{T}_e)}+\tan^{-1}\left(\frac{2k_\mu}{q_{\mathrm{TF}}}\right)\right], \qquad \text{(S25)}$$

and the coefficients $A_{0,1,2}(\mathcal{E},\tilde{T}_e)$ are given by

$$\begin{aligned}
A_{n=0,1,2}(\mathcal{E},\tilde{T}_e)&=\frac{2k_\mu(\tilde{T}_e)}{k(\mathcal{E})}\left[\frac{2k_\mu(\tilde{T}_e)q_{\mathrm{TF}}(\tilde{T}_e)}{4k_\mu^2(\tilde{T}_e)+q_{\mathrm{TF}}^2(\tilde{T}_e)}+\tan^{-1}\left(\frac{2k_\mu(\tilde{T}_e)}{q_{\mathrm{TF}}(\tilde{T}_e)}\right)\right]^{-1}\\
&\quad (1+2C\mu(\tilde{T}_e))^{-3}\iint\limits_{0<\mathcal{E}_1,\mathcal{E}_2,\mathcal{E}+\mathcal{E}_1-\mathcal{E}_2<\mathcal{E}_{\max}} d\mathcal{E}_1 d\mathcal{E}_2\left(-\frac{1}{2}\right)^n\tanh^n\left(\frac{\mathcal{E}_1-\mathcal{E}_2}{2k_B\tilde{T}_e}\right)\\
&\quad \left[\cosh\left(\frac{\mathcal{E}_1-\mu(\tilde{T}_e)}{2k_B\tilde{T}_e}\right)\cosh\left(\frac{\mathcal{E}_2-\mu(\tilde{T}_e)}{2k_B\tilde{T}_e}\right)\cosh\left(\frac{\mathcal{E}_1-\mathcal{E}_2}{2k_B\tilde{T}_e}\right)\right]^{-1}\\
&\quad \left[\frac{q q_{\mathrm{TF}}}{q^2+q_{\mathrm{TF}}^2}+\tan^{-1}\left(\frac{q}{q_{\mathrm{TF}}}\right)\right]_{q_{\min}}^{q_{\max}}(1+2C\mathcal{E}_1)(1+2C\mathcal{E}_2)(1+2C(\mathcal{E}+\mathcal{E}_1-\mathcal{E}_2)). \qquad \text{(S26)}
\end{aligned}$$

This result is a generalization of Equation (19) in [10], which was derived for moderate rises in the electron temperature and was not tested vs. the rigorous calculation for strong illumination.

Figure S1(a) compares the *e-e* relaxation rate calculated using the exact results (Equation (S19)) and the *generalized* Fermi-liquid approximation (S24) for the examples used in the main text. The discrepancy near the chemical potential arises from the thermal approximation introduced in Equation (S21), whereas deviations at energies farther from the chemical potential are primarily due to the power series expansion in $(\mathcal{E}-\mu(\tilde{T}_e))$ used in Equation (S23). Note that the linear term also causes asymmetry with respect to the chemical potential μ.

We emphasize that the key deviation from the standard FLT lies in our deliberate retention of a finite lower integration limit in Equation (S26), corresponding to the conduction band minimum, rather than extending it to $-\infty$ as is typically done. This modification is physically motivated by the fact that the chemical potential lies close to the lower edge of the conduction band, making the $-\infty$ extension inappropriate. As a consequence of this finite integration domain, the coefficients A_0 and A_2 are reduced from their standard Fermi-liquid values (π^2 and 1, respectively), and they decrease further with increasing $\tilde{T}_e$

(Figs. S1(b) and (d)). Additionally, a linear term emerges in Equation (S24), which would otherwise vanish under the standard Fermi-liquid approximation (Figure S1(c)) [2]. This linear term partially suppresses the standard Fermi-liquid form, as its coefficient A_1 is negative and becomes increasingly negative with rising $\tilde{T}_e$ (Figure S1(c)). Consequently, $\eta_{\text{e-e}}$ is reduced for energies $\mathcal{E} > \mu(\tilde{T}_e)$. Since $\mu(\tilde{T}_e)$ decreases with increasing $\tilde{T}_e$, a larger portion of the electron distribution lies above $\mu(\tilde{T}_e)$, making the suppressive effect of the linear term more pronounced at higher $\tilde{T}_e$.

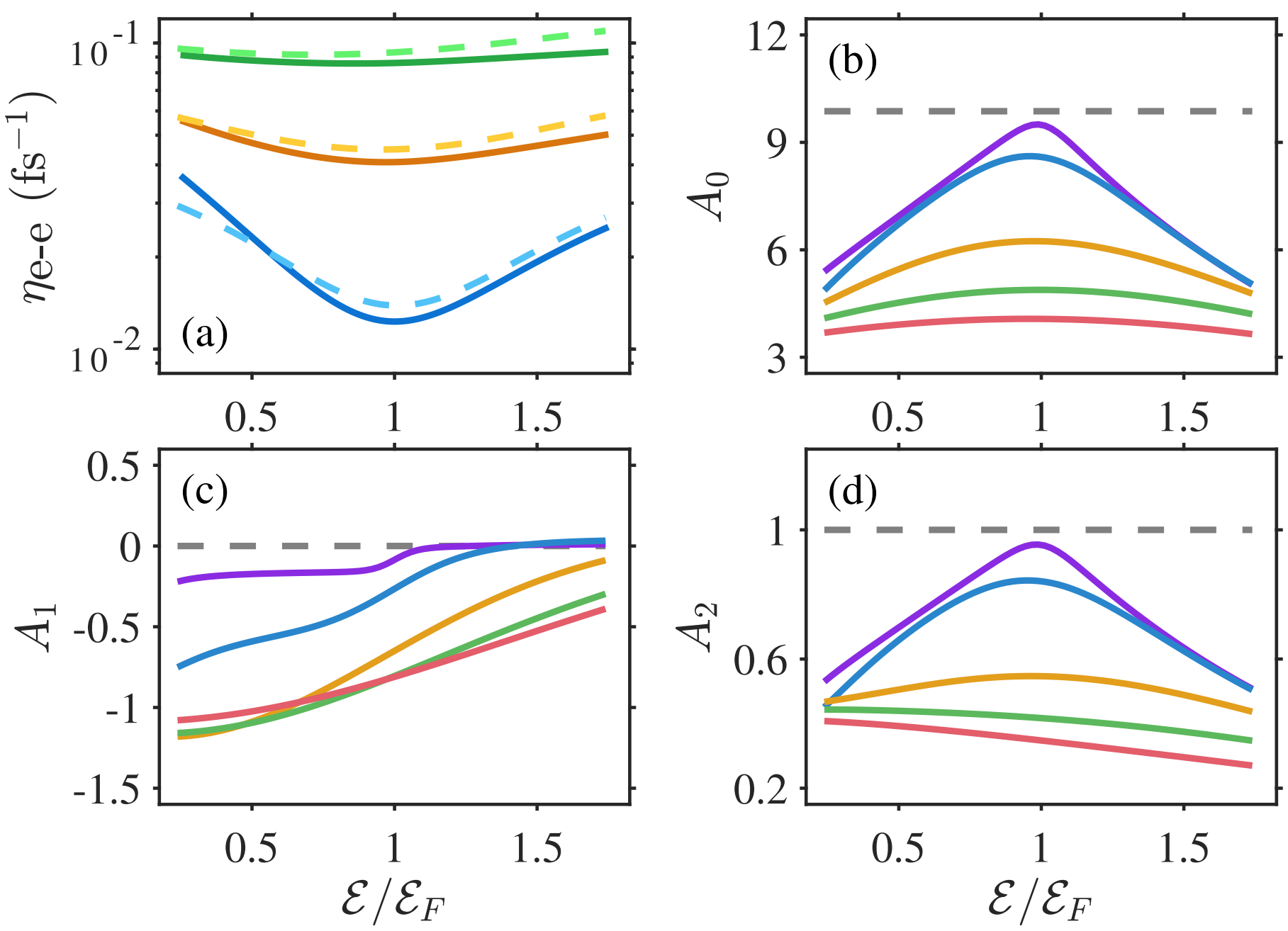

Figure S1: (a) The e-e relaxation rate for $E_0 = 0.6$ V/nm (blue lines), $E_0 = 1.2$ V/nm (orange lines), and $E_0 = 1.8$ V/nm (green lines) at $t = 5$ fs. The solid and dashed lines represent the exact results (Equation (S19)) and the Fermi-liquid approximation (S24), respectively. The coefficients (b) A_0, (c) A_1, and (d) A_2 (Equation (S26)) as a function of electron energy $\mathcal{E}$ for $\tilde{T}_e = 300$ K (purple lines), 1000 K (blue lines), 3000 K (orange lines), 5000 K (green lines), and 7000 K (red lines). The black dashed lines represent the corresponding values (π^2, 0, and 1 in (a), (b), and (c), respectively) in the conventional Fermi-liquid formulation.

S3 Supplemental figures

Figure S2 plots the dynamics of the excited electron density, number density of electrons per unit volume per unit energy, defined as

$$\left[f(\mathcal{E},t) - f^{\text{FD}}(\mathcal{E},T_e = 300\text{K})\right] \frac{\rho_e(\mathcal{E})}{\rho_e(\mathcal{E}_F)}. \tag{S27}$$

Figure S3 plots the contribution to the permittivity change from stimulated emission.

[2] Note that Equation (19) in Ref. [10] also had a linear correction, but it can be shown to be at least 10 times smaller than the leading order terms near the chemical potential.

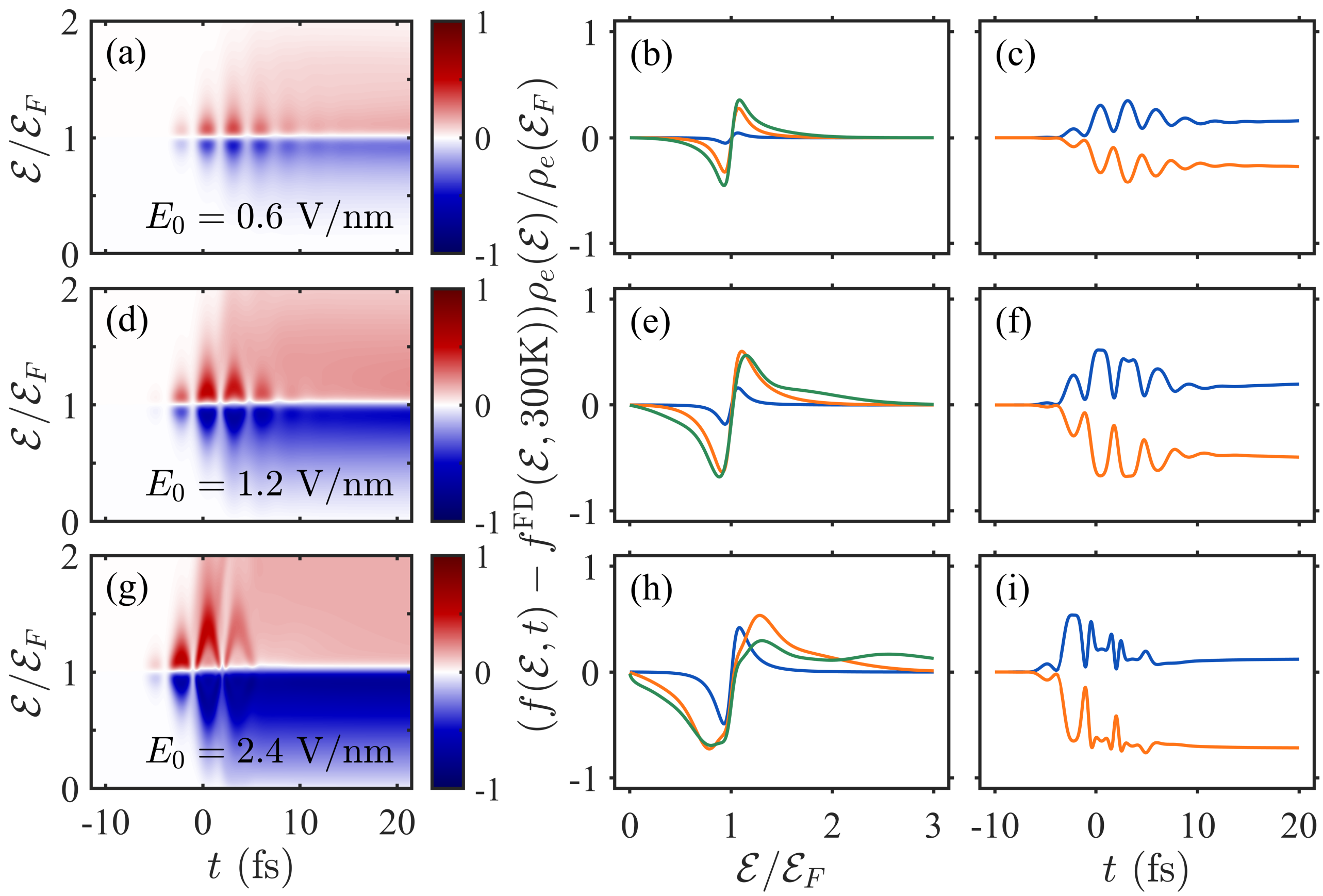

Figure S2: (a) An energy-time map of the excited electron density dynamics, $\left(f(\mathcal{E},t) - f^{\mathrm{FD}}(\mathcal{E}, T_e = 300\mathrm{K})\right)\rho_e(\mathcal{E})/\rho_e(\mathcal{E}_F)$ (b) fixed time snapshots of the excited electron density at $t = -2.5$ femtoseconds (blue), 0 femtoseconds (orange), and 5 femtoseconds (green), and (c) the excited electron density dynamics at $\mathcal{E} = \mathcal{E}_F \pm \hbar\omega_0/8$ (blue and orange) for $E_0 = 0.6$ V/nm. (d)-(f) and (g)-(i) show similar data as (a)-(c) for $E_0 = 1.2$ V/nm and 2.4 V/nm, respectively.

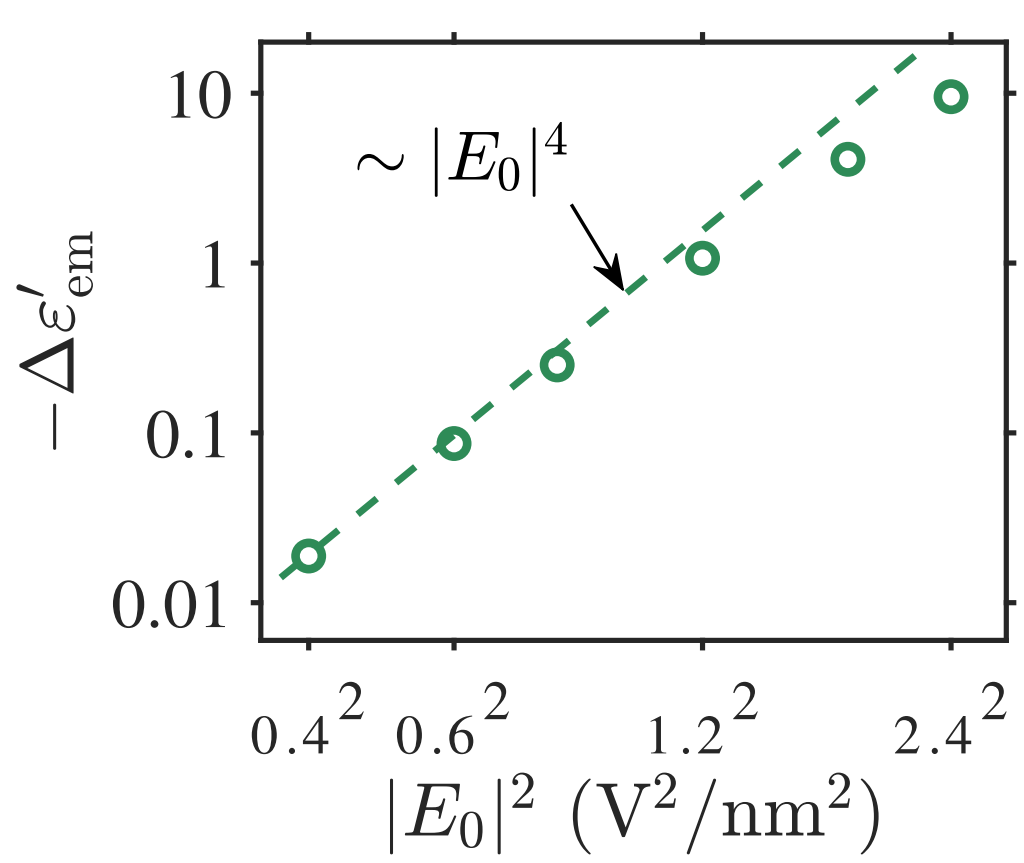

Figure S3: The (negative) contribution to the permittivity change due to stimulated emission (green unfilled circles). The dashed line represents the $|E_0|^4$ fit to the permittivity change.

References

[1] I. W. Un, S. Sarkar, Y. Sivan, *Phys. Rev. B* **2026**, *113* 165411.

[2] A. O. Govorov, H. Zhang, Y. K. Gun'ko, *J. Phys. Chem. C* **2013**, *117* 16616.

[3] J. B. Khurgin, U. Levy, *ACS Photonics* **2020**, *7* 547.

[4] D. W. Snoke, *Solid State Physics: Essential Concepts*, Pearson/Addison-Wesley, Boston, MA, 2nd edition, **2020**.

[5] G. Grosso, G. Parravicini, *Solid State Physics*, Academic Press, London, 2nd edition, **2014**.

[6] N. D. Fatti, C. Voisin, M. Achermann, S. Tzortzakis, D. Christofilos, F. Valleé, *Phys. Rev. B* **2000**, *61* 16956.

[7] I. W. Un, S. Sarkar, Y. Sivan, *Phys. Rev. Applied* **2023**, *19* 044043.

[8] M. Tokman, M. Erukhimova, *J. Lumin.* **2013**, *137* 148.

[9] M. Erukhimova, Y. Wang, M. Tokman, A. Belyanin, *Phys. Rev. B* **2020**, *102* 235103.

[10] S. Sarkar, I. W. Un, Y. Sivan, *Phys. Rev. Applied* **2023**, *19* 014005.

[11] S. Sarkar, I. W. Un, Y. Sivan, Comparative study of models of optically-induced electron non-equilibrium in drude metals, **2024**.

[12] X. Liu, J. Park, J.-H. Kang, H. Yuan, Y. Cui, H. Y. Hwang, M. L. Brongersma, *Appl. Phys. Lett.* **2014**, *105*, 18 181117.

[13] H. Wang, K. Du, C. Jiang, Z. Yang, L. Ren, W. Zhang, S. J. Chua, T. Mei, *Phys. Rev. Applied* **2019**, *11* 064062.

[14] V. Brinzari, D. Nika, I. Damaskin, B. Cho, G. Korotcenkov, *Physica E* **2016**, *81* 49.

[15] P. Coleman, *Introduction to many body physics*, Cambridge University Press, Cambridge, **2015**.
